# Acamprosate for alcohol dependence

**DOI:** 10.1590/S1516-31802010000600014

**Published:** 2010-12-02

## Abstract

**BACKGROUND:**

Alcohol dependence is among the main leading health risk factors in most developed and developing countries. Therapeutic success of psychosocial programs for relapse prevention is moderate, but could potentially be increased by an adjuvant treatment with the glutamate antagonist acamprosate.

**OBJECTIVE:**

To determine the effectiveness and tolerability of acamprosate in comparison to placebo and other pharmacological agents.

**CRITERIA FOR CONSIDERING STUDIES FOR THIS REVIEW:**

We searched the Cochrane Drugs and Alcohol Group (CDAG) Specialized Register, PubMed, Embase and CINAHL in January 2009 and inquired manufacturers and researchers for unpublished trials.

**SELECTION CRITERIA:**

All double-blind randomised controlled trials (RCTs) which compare the effects of acamprosate with placebo or active control on drinking-related outcomes.

**DATA COLLECTION AND ANALYSIS:**

Two authors independently extracted data. Trial quality was assessed by one author and cross-checked by a second author. Individual patient data (IPD) meta-analyses were used to verify the primary effectiveness outcomes.

**MAIN RESULTS:**

24 RCTs with 6915 participants fulfilled the criteria of inclusion and were included in the review. Compared to placebo, acamprosate was shown to significantly reduce the risk of any drinking RR 0.86 (95% CI 0.81 to 0.91); NNT 9.09 (95% CI 6.66 to 14.28) and to significantly increase the cumulative abstinence duration MD 10.94 (95% CI 5.08 to 16.81), while secondary outcomes (gamma-glutamyltransferase, heavy drinking) did not reach statistical significance. Diarrhea was the only side effect that was more frequently reported under acamprosate than placebo RD 0.11 (95% 0.09 to 0.13); NNTB 9.09 (95% CI 7.69 to 11.11). Effects of industry-sponsored trials RR 0.88 (95% 0.80 to 0.97) did not significantly differ from those of non-profit funded trials RR 0.88 (95% CI 0.81 to 0.96). In addition, the linear regression test did not indicate a significant risk of publication bias (P = 0.861).

**AUTHORS’ CONCLUSIONS:**

Acamprosate appears to be an effective and safe treatment strategy for supporting continuous abstinence after detoxification in alcohol dependent patients. Even though the sizes of treatment effects appear to be rather moderate in their magnitude, they should be valued against the background of the relapsing nature of alcoholism and the limited therapeutic options currently available for its treatment

**FURTHER INFORMATION:** Centro Cochrane do Brasil Rua Pedro de Toledo, 598 Vila Clementino — São Paulo (SP) — Brasil CEP 04039-001 Tel. (+55 11) 5579-0469/5575-2970 http://www.centrocochranedobrasil.org.br/

**For Latin America and Caribbean, the full text of the review is freely available from:** http://cochrane.bvsalud.org/cochrane/main.php?lang=pt&lib=COC

**For other regions, the abstract is available from:** http://onlinelibrary.wiley.com/o/cochrane/clsysrev/articles/CD004332/frame.html

## COMMENTS

This paper demonstrates the results obtained using acamprosate in a double-blind study conducted in 24 centers, involving Europe, United States, South Korea, Australia and Brazil.^1^ Over 6,000 subjects were investigated, and they showed good tolerance to the drug and a statistically significant reduction in the risk of immediate recurrence of alcohol use. However, late recurrence was not avoided. This is therefore a further therapeutic resource with a low rate of adverse events, but its superiority in relation to other drugs used for the same purpose needs to be proven.

The development of new drugs for treating alcohol dependence is of inestimable value because of the high incidence and prevalence of the disease and comorbidities deriving from it, which raise the morbidity and mortality rates, especially among young people. New drugs need to present favorable pharmacokinetic characteristics (the way in which the body absorbs, metabolizes and binds to proteins and eliminates the drug) and pharmacodynamic characteristics (the action of the drug on the body, its effect on receptors and its interactions with other drugs), and preferably need to be affordable.
